# LRRK2 integrates Rab and GABARAP interactions to sense and respond to distinct lysosomal stresses

**DOI:** 10.1101/2025.11.19.689251

**Published:** 2025-11-19

**Authors:** Devin Clegg, Amanda Bentley-DeSousa, Agnes Roczniak-Ferguson, Shawn M. Ferguson

**Affiliations:** 1 Departments of Cell Biology, Yale University School of Medicine, New Haven, Connecticut 06510, USA.; 2 Department of Neuroscience, Yale University School of Medicine, New Haven, Connecticut 06510, USA.; 3 Program in Cellular Neuroscience, Neurodegeneration and Repair, Yale University School of Medicine, New Haven, Connecticut 06510, USA.; 4 Wu Tsai Institute, Yale University School of Medicine, New Haven, Connecticut 06510, USA.; 5 Kavli Institute for Neuroscience, Yale University School of Medicine, New Haven, Connecticut 06510, USA.; 6 Aligning Science Across Parkinson’s (ASAP) Collaborative Research Network, Chevy Chase, MD, 20815, USA.

## Abstract

Increased activity of leucine-rich repeat kinase 2 (LRRK2) is an important risk factor for Parkinson’s disease. LRRK2 localizes to lysosomal membranes, and changes in lysosome physiology are emerging as key regulators of its activation, yet the mechanisms by which distinct perturbations engage this kinase remain unclear. Analysis of osmotic and membrane-integrity challenges revealed that LRRK2 integrates multiple upstream cues through parallel interactions with Rab GTPases and GABARAP. Manipulations that caused lysosome enlargement, including inhibition of PIKfyve, showed that osmotic swelling leads to the accumulation of multiple Rabs on lysosomes and Rab-dependent LRRK2 activation independently of GABARAP. In contrast, under conditions of lysosome deacidification, CASM-dependent lipidation of GABARAP creates a platform that cooperates with Rabs in LRRK2 activation. These findings demonstrate how LRRK2 interprets perturbations of lysosome function through a combination of Rab- and GABARAP-dependent mechanisms, providing a framework for understanding both normal physiological regulation and pathological dysregulation in Parkinson’s disease.

## Introduction

Mutations that increase the activity of leucine-rich repeat kinase 2 (LRRK2) are a major cause of familial Parkinson’s disease, and common LRRK2 variants also elevate risk for sporadic Parkinson’s disease (Alessi & Pfeffer, 2024; Bentley-DeSousa, Clegg, et al., 2025; Paisan-Ruiz et al., 2004; Rocha et al., 2022; Zimprich et al., 2004). Pathogenic mutations in other Parkinson’s disease genes, including Rab32 and VPS35, also enhance LRRK2 signaling, underscoring the importance of understanding how upstream cellular processes regulate LRRK2 activation (Gustavsson et al., 2024; Hop et al., 2024; McCarron et al., 2024; Mir et al., 2018). Defining these mechanisms is essential for explaining how inappropriate LRRK2 activation contributes to Parkinson’s disease as well as for understanding physiological functions of LRRK2.

LRRK2 activity is strongly stimulated by lysosomal stress, leading to its recruitment to lysosomal membranes and activation of its kinase activity (Bentley-DeSousa et al., 2024; Bentley-DeSousa, Roczniak-Ferguson, et al., 2025; Bonet-Ponce et al., 2020; Eguchi et al., 2024; Kalogeropulou et al., 2020; McCarron et al., 2024). Recent work revealed that lysosomal membrane damage and other triggers of deacidification activate LRRK2 through the process known as conjugation of ATG8 proteins to single membranes (CASM), which enables GABARAP proteins to recruit and activate LRRK2 (Bentley-DeSousa et al., 2024; Bentley-DeSousa, Roczniak-Ferguson, et al., 2025; Eguchi et al., 2024). While this CASM–GABARAP pathway explains how lysosome deacidification signals to LRRK2, it remains unclear whether other physiologically relevant lysosomal stresses, such as osmotic imbalances, engage LRRK2 through similar or distinct mechanisms.

Multiple Rab GTPases have been shown to bind directly to LRRK2 and facilitate its recruitment to membranes (Alessi & Pfeffer, 2024; Dhekne et al., 2023; Purlyte et al., 2018; Vides et al., 2022; Wang et al., 2023). Yet, how Rab-dependent and GABARAP-dependent mechanisms are coordinated at lysosomes, and which specific forms of lysosomal stress rely on one mode of recruitment over the other, remains unresolved. This gap limits our understanding of how LRRK2 interprets the diverse stress signatures that arise both during normal lysosome homeostasis and under pathological conditions linked to Parkinson’s disease. With respect to Parkinson’s disease, dual roles of Rabs 29 and 32 as LRRK2 activators and Parkinson’s disease risk genes highlights the broader importance of defining how and when such proteins are engaged in LRRK2 regulation (Gustavsson et al., 2024; Hop et al., 2024).

The lipid kinase PIKfyve synthesizes phosphatidylinositol-3,5-bisphosphate [PI(3,5)P_2_], a low-abundance signaling lipid essential for endolysosomal membrane dynamics and ion homeostasis (Gary et al., 1998; Leray et al., 2022; Rivero-Rios & Weisman, 2022). Inhibition of PIKfyve depletes PI(3,5)P_2_, thereby relieving inhibition of the chloride/proton (Cl^−^/H^+^) antiporter known as ClC-7 and causing lysosomal chloride influx and pronounced osmotic swelling (Cao et al., 2023; Leray et al., 2022; Nicoli et al., 2019; Wu et al., 2025). The result is massive enlargement of lysosomes that involves both a coalescence of existing lysosomes due to an imbalance in lysosome fission and fusion as well as the delivery of new membrane to lysosomes via contact sites with the endoplasmic reticulum (Choy et al., 2018; Yang et al., 2025). Because PIKfyve inhibition potently induces lysosomal enlargement and has been linked to increased LRRK2 activity via unknown mechanisms, this perturbation provides a tractable model for dissecting the contribution of distinct lysosome stresses to LRRK2 regulation (McCarron et al., 2024).

Here, we used the PIKfyve–ClC-7 pathway and complementary manipulations of lysosomal osmotic balance to define how swelling of lysosomes regulates LRRK2. We show that PIKfyve inhibition activates LRRK2 through a mechanism that sequentially requires ClC-7, lysosomal swelling, and LRRK2-Rab GTPase interactions, but which is independent of CASM–GABARAP. Preventing lysosomal swelling abolished LRRK2 activation by PIKfyve inhibition, whereas directly inducing lysosome osmotic stress was sufficient to trigger an increase in LRRK2 activity independent of Pikfyve and ClC-7. Moreover, we demonstrate that LRRK2-Rab interactions are essential for activation by both swelling and CASM-dependent stimuli, identifying Rabs as a unifying hub through which diverse lysosomal stress pathways converge on LRRK2. Together, these findings provide a framework for understanding how LRRK2 integrates multiple upstream signals, mechanical, metabolic, and damage-related, to control its homeostatic and pathogenic roles.

## Results

### PIKfyve inhibition activates LRRK2 and drives its recruitment to lysosomes

**Figure 1.**
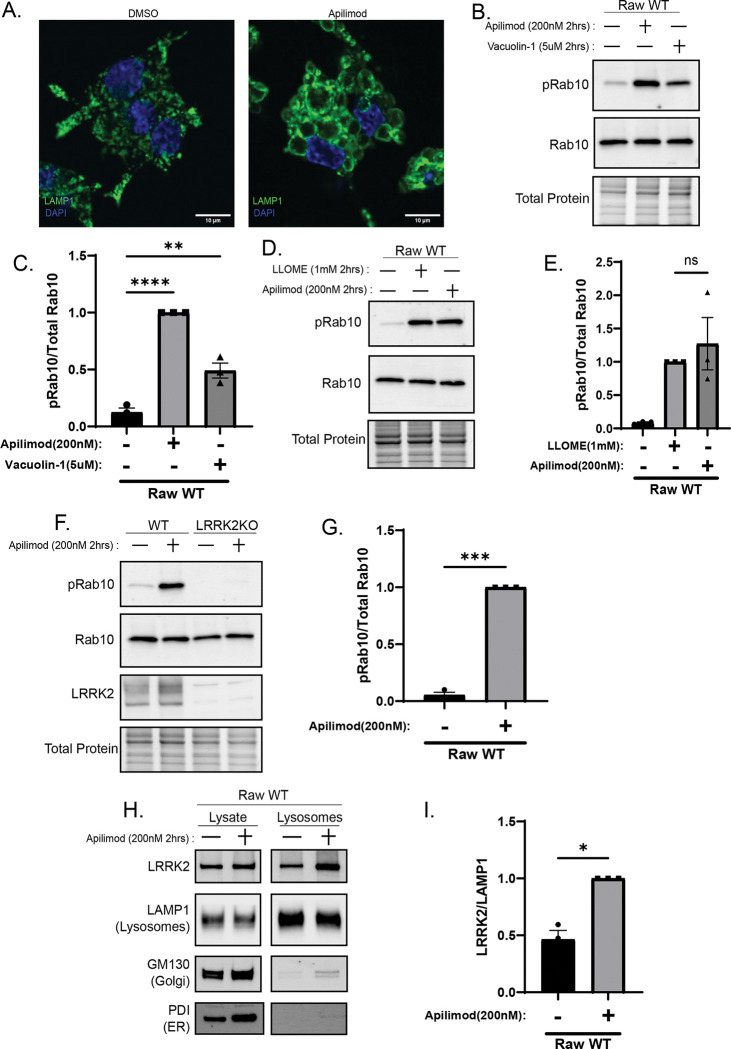
PIKfyve inhibition activates LRRK2 and drives its recruitment to lysosomes. (A) Spinning disk confocal immunofluorescence imaging of wild-type Raw 264.7 cells treated with DMSO or apilimod (200nM, 2 h) showing enlarged lysosomes (LAMP1, green; DAPI). (B) Immunoblot analysis of cells treated with apilimod (200nM, 2 h) or vacuolin-1 (5uM, 2 h) showing increased phosphorylation of Rab10 at T73 (pRab10 [T73]). (C) Quantification of pRab10 (T73) after apilimod or vacuolin-1 treatment (mean ± SEM; n = 3, one-way ANOVA, P< 0.0001). (D) Immunoblot of wild-type Raw 264.7 cells treated with LLOME (1mM, 2 h) or apilimod (200nM, 2 h). (E) Quantification of pRab10 (T73) after PIKfyve inhibition or LLOME (mean ± SEM; n = 3, one-way ANOVA, P< 0.0235). (F) Immunoblot of wild-type and *Lrrk2* KO Raw 264.7 cells treated with apilimod (200nM, 2 h). (G) Quantification of pRab10 (T73) in wild-type and *Lrrk2* KO cells (mean ± SEM; n = 3, two-tailed unpaired Welch’s t test, P< 0.001). (H) Immunoblot of lysates and purified lysosomes after apilimod(200nM, 2 h) treatment showing increased LRRK2 at lysosomes. (I) Quantification of lysosomal LRRK2 (mean ± SEM; n = 3, two-tailed unpaired Welch’s t test, P= 0.020).

Because depletion of PI(3,5)P_2_ through inhibition of the lipid kinase PIKfyve acutely causes robust lysosomal swelling, we used this perturbation as a tractable model to investigate how osmotic changes influence LRRK2 regulation (Rivero-Rios & Weisman, 2022). Consistent with prior studies across multiple cell types, we observed that pharmacological inhibition of PIKfyve with apilimod caused pronounced lysosome enlargement in the Raw264.7 mouse macrophage cell line, (Fig. 1A)(Choy et al., 2018; Compton et al., 2016; Gayle et al., 2017; Rivero-Rios & Weisman, 2022; Yang et al., 2025). This was accompanied by a robust increase in Rab10 phosphorylation at threonine 73, a well-established LRRK2 substrate, which was similarly induced by additional PIKfyve inhibitors including vacuolin-1 and APY0201 (Fig. 1B–C, S1A) (Steger et al., 2016). Elevated phosphorylation of additional LRRK2 substrates, Rab8 (T72) and Rab12 (S106), further confirmed activation of LRRK2 kinase activity (Fig. S1E–H). The absence of Rab10 phosphorylation in apilimod-treated LRRK2-knockout (KO) cells and its loss upon treatment with the LRRK2 inhibitor MLi-2 independently demonstrated the LRRK2 dependence of this Rab phosphorylation (Fig. 1F–G, S1C). Comparable LRRK2 activation in response to PIKfyve inhibition was observed in mouse embryonic fibroblasts, BV-2 microglia, and human iPSC-derived microglia (Fig. S1I–N), indicating conservation of the effect across species and cell types. Immunoblot analysis of purified lysosomes revealed a marked accumulation of LRRK2 at lysosomes following PIKfyve inhibition (Fig. 1H–I), consistent with a close relationship between LRRK2 lysosomal localization and activation (Bentley-DeSousa, Clegg, et al., 2025).

### ClC-7 is required for LRRK2 activation downstream of PIKfyve inhibition

**Figure 2.**
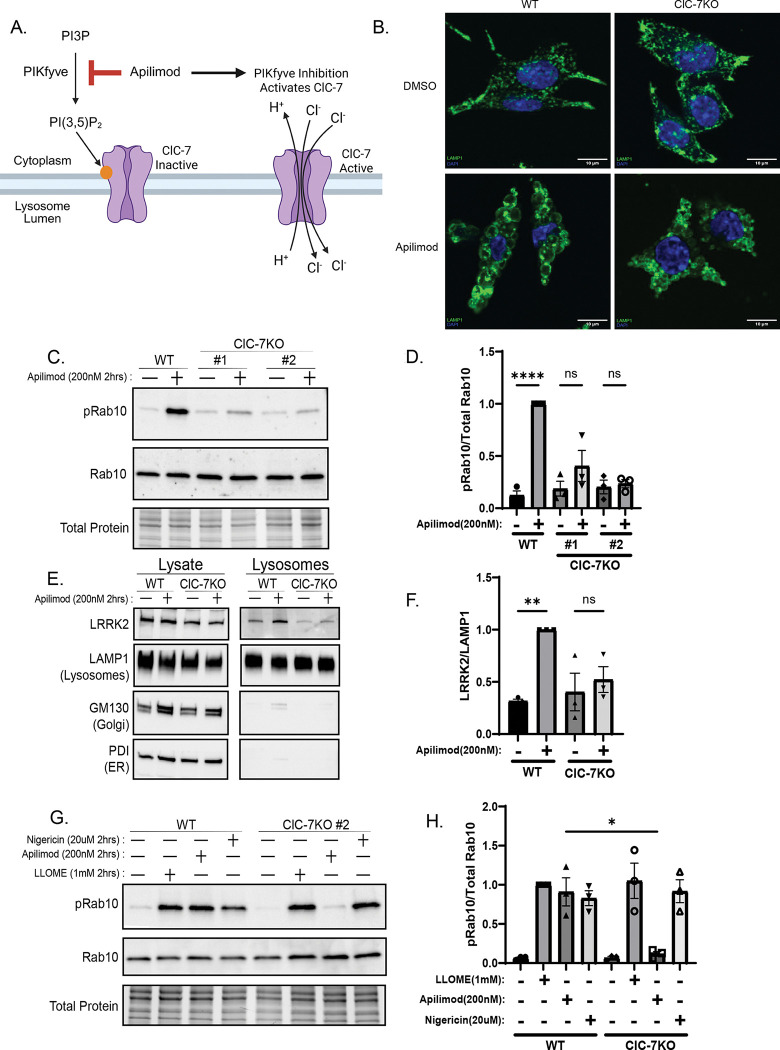
ClC-7 is required for LRRK2 activation by PIKfyve inhibition. (A) Schematic illustrating how PIKfyve inhibition depletes PI(3,5)P_2_, relieves ClC-7 inhibition, and promotes lysosomal chloride influx and swelling, Created in https://BioRender.com. (B) Immunofluorescence imaging of wild-type and *Clcn7* KO Raw 264.7 cells treated with DMSO or apilimod (200nM, 2 h) (LAMP1, green; DAPI). (C) Immunoblot analysis of wild-type and *Clcn7* KO cells treated with DMSO or apilimod and probed for pRab10 (T73). (D) Quantification of pRab10 (T73) after apilimod in *Clcn7* KO cells (mean ± SEM; n = 3, one-way ANOVA, P< 0.0001). (E) Immunoblot of lysates and purified lysosomes from wild-type and *Clcn7* KO cells after apilimod (200nM, 2 h). (F) Quantification of lysosomal LRRK2 (mean ± SEM; n = 3, one-way ANOVA, P= 0.0095). (G) Immunoblot of wild-type and *Clcn7* KO cells treated with apilimod (200nM, 2 h) or CASM activators (LLOME, nigericin; 1mM, 20uM for 2 h) and probed for pRab10 (T73). (H) Quantification of pRab10 (T73) after apilimod or CASM activators (mean ± SEM; n = 3, two-tailed unpaired Welch’s t test, P= 0.044).

To identify molecular mechanisms linking PIKfyve inhibition to LRRK2 activation, we next focused on ClC-7, a lysosomal chloride-proton antiporter whose activity is strongly enhanced when PI(3,5)P_2_ levels fall and a major mediator of the cellular effects of PIKfyve inhibition (Gayle et al., 2017; Leray et al., 2022). In ClC-7-KO cells, PIKfyve inhibition failed to induce the characteristic increase in lysosome size and did not elevate Rab10 phosphorylation (Fig. 2B–D). LRRK2 accumulation at lysosomes was likewise impaired (Fig. 2E–F). This requirement was specific: LRRK2 activation by LLOME or nigericin, which trigger the CASM–GABARAP pathway, remained intact in ClC-7-KO cells (Fig. 2G–H; Bentley-DeSousa, Roczniak-Ferguson, et al., 2025). TRPML1 is a lysosomal cation channel that is positively regulated by PI(3,5)P2. However, pharmacological inhibition of TRPML1 had no significant effect on LRRK2 activity, supporting a model in which PIKfyve inhibition activates LRRK2 primarily by relieving ClC-7 inhibition (Fig. S2B-C)(Dong et al., 2010).

### Lysosomal swelling drives Rab accumulation and LRRK2 activation

**Figure 3.**
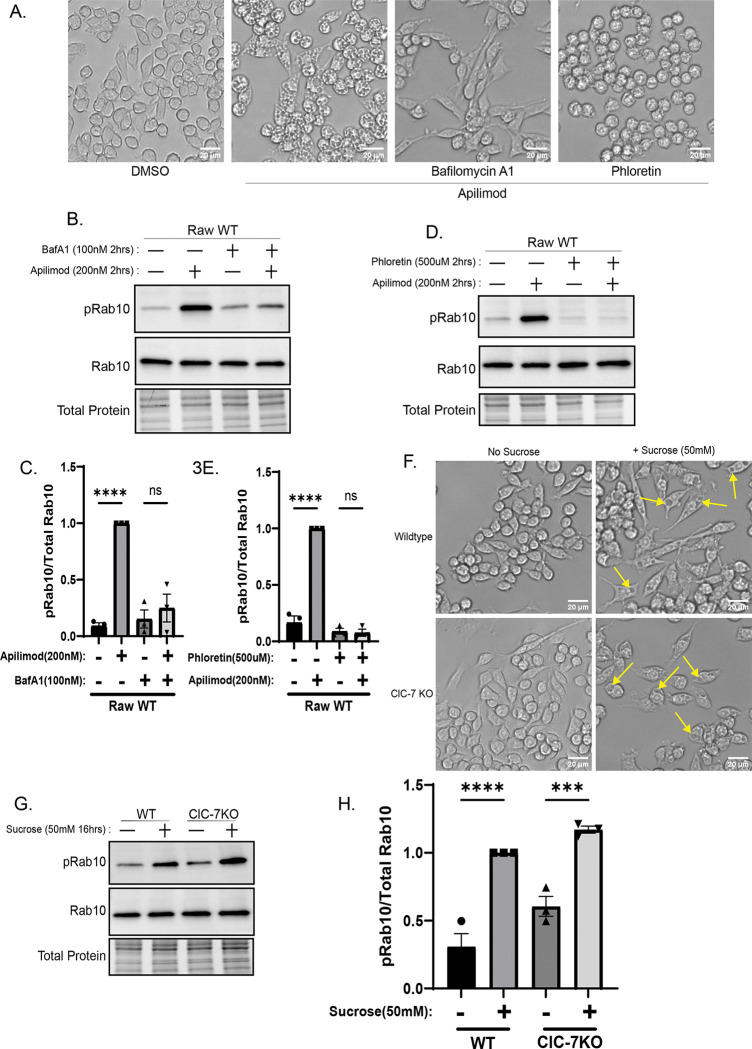
Lysosomal swelling drives LRRK2 activation. (A) Bright-field images of Raw 264.7 cells treated with apilimod (200nM, 2 h) and pretreated with bafilomycin A1 or phloretin (100nM, 500mM for 30 min). (B) Immunoblot analysis of cells pretreated with bafilomycin A1 before apilimod and probed for pRab10 (T73). (C) Quantification of pRab10 (T73) after bafilomycin A1 pretreatment (mean ± SEM; n = 3, one-way ANOVA, P< 0.0001). (D) Immunoblot analysis of cells pretreated with phloretin before apilimod (probed for pRab10 [T73]). (E) Quantification of pRab10 (T73) after phloretin pretreatment (mean ± SEM; n = 3, one-way ANOVA, P< 0.0001). (F) Bright-field images of wild-type and *Clcn7* KO Raw 264.7 cells incubated in medium containing 50 mM sucrose for 16 h, yellow arrows pointing at enlarged lysosomes. (G) Immunoblot analysis of cells after sucrose treatment (50 mM sucrose for 16 h) probed for pRab10 (T73). (H) Quantification of pRab10 (T73) after sucrose exposure (mean ± SEM; n = 3, one-way ANOVA, P< 0.0001). Together, these results demonstrate that osmotic swelling of lysosomes is both necessary and sufficient for LRRK2 activation.

To determine whether lysosome swelling is required for LRRK2 activation, we pharmacologically prevented enlargement using two complementary approaches. Inhibition of the V-ATPase with bafilomycin A1 prevented apilimod-induced lysosome enlargement and suppressed LRRK2 activation (Fig. 3A–C). Likewise, treatment with phloretin, an inhibitor of aquaporins and other transporters, blocked both lysosome swelling and the associated increase in LRRK2 kinase activity (Fig. 3A, D–E)(Yang et al., 2025). To test whether swelling alone is sufficient to activate LRRK2, we induced osmotic stress independent of PIKfyve inhibition. Addition of sucrose to the culture medium causes the formation of enlarged lysosomes (“sucrosomes”)(Bright et al., 2016; Cai et al., 2024). This occurs because sucrose is taken up by endocytosis and accumulates in lysosomes as they lack the invertase enzyme that breaks down sucrose. In both WT and ClC-7 KO cells, sucrose caused both lysosome enlargement and LRRK2 activation (Fig. 3F-H). These results collectively demonstrate that osmotic swelling of lysosomes, rather than other downstream effects of PIKfyve inhibition or ClC-7 activation, is sufficient to trigger LRRK2 activation.

### Rab interactions are required for LRRK2 recruitment and activation at swollen lysosomes

**Figure 4.**
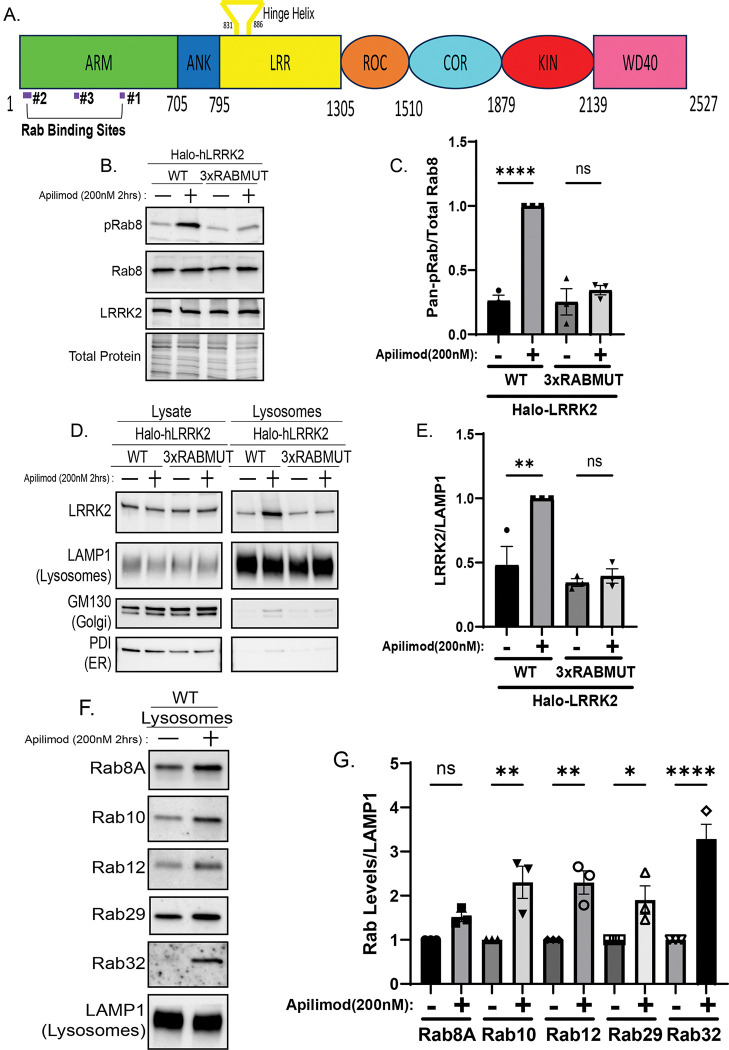
Rab GTPase interactions are required for LRRK2 recruitment and activation. (A) Schematic of LRRK2 showing its three Rab-binding sites. (B) Immunoblot analysis of Raw 264.7 cells stably expressing wild-type or Rab-binding-deficient (3×Rab) LRRK2 treated with apilimod (200nM, 2 h) and probed for pRab10 (T73). (C) Quantification of pRab10 (T73) in wild-type and 3×Rab cells (mean ± SEM; n = 3, one-way ANOVA, P< 0.0001). (D) Immunoblot analysis of lysates and purified lysosomes showing recruitment of LRRK2 to lysosomes after apilimod treatment. (E) Quantification of lysosomal LRRK2 (mean ± SEM; n = 3, one-way ANOVA, P= 0.0013). (F) Immunoblot analysis of lysosomal Rab proteins—Rab8A, Rab10, Rab12, Rab29, and Rab32—after apilimod. (G) Quantification of lysosomal Rab accumulation (mean ± SEM; n = 3, one-way ANOVA, P< 0.0001).

Having established that lysosomal osmotic swelling is sufficient to activate LRRK2, we next sought to define molecular mechanisms that couple this biophysical signal to LRRK2 recruitment and kinase activation. Although multiple Rab-binding sites on LRRK2 have been defined, the upstream signals that regulate Rab-dependent activation of LRRK2 remain poorly understood (Alessi & Pfeffer, 2024; Bentley-DeSousa, Clegg, et al., 2025; Dhekne et al., 2023; Vides et al., 2022; Wang et al., 2023)(Fig. 4A and S3A). Because Rab GTPases mediate LRRK2 membrane recruitment, we tested their requirement in swelling-induced activation. LRRK2-KO Raw264.7 cells were complemented with either wild-type LRRK2 or a Rab-binding-deficient mutant (K17/K18A + E240R + K439E, “3×Rab”) lacking the three major Rab-interaction sites that collectively support binding to Rab8A, 8B, 10, 12, 29, 32, and 38 (Alessi & Pfeffer, 2024; Bentley-DeSousa, Clegg, et al., 2025). The LRRK2 3×Rab mutant failed to phosphorylate Rab substrates or accumulate on lysosomes following PIKfyve inhibition (Fig. 4B–E). Meanwhile, endogenous Rab8A, Rab10, Rab12, Rab29, and Rab32 all increased in abundance on lysosomes after PIKfyve inhibition (Fig. 4F–G), establishing a correlation between swelling, Rab accumulation, and LRRK2 recruitment. Inhibition of LRRK2 with MLi-2 abolished Rab phosphorylation but did not prevent either Rab or LRRK2 accumulation at lysosomes. This indicates that Rab accumulation precedes LRRK2 recruitment and activation (Fig. S3B–F). Thus, osmotic swelling promotes Rab enrichment on lysosomal membranes, defining a physiological context in which Rab-dependent recruitment and activation of LRRK2 occurs.

### Distinct stress pathways converge on Rab and GABARAP-dependent LRRK2 activation

**Figure 5.**
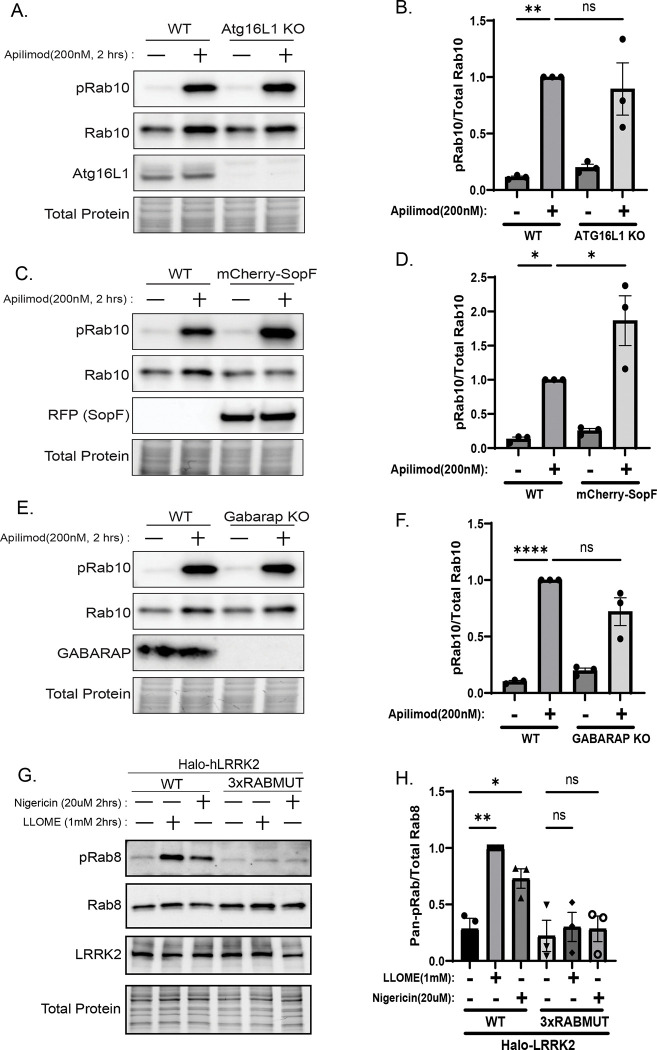
Distinct pathways converge on Rab-dependent LRRK2 activation. (A) Immunoblot analysis of wild-type and *Atg16l1* KO cells treated with apilimod (200nM, 2 h) and probed for pRab10 (T73). (B) Quantification of pRab10 (T73) in wild-type and *Atg16l1* KO cells (mean ± SEM; n = 3, one-way ANOVA, P= 0.0011). (C) Immunoblot of wild-type and mCherry-SopF–expressing cells treated with apilimod (200nM, 2 h) and probed for pRab10 (T73). (D) Quantification of pRab10 (T73) in wild-type and SopF cells (mean ± SEM; n = 3, one-way ANOVA, P= 0.0005). (E) Immunoblot of wild-type and *Gabarap* KO cells treated with apilimod (200nM, 2 h). (F) Quantification of pRab10 (T73) in wild-type and *Gabarap* KO cells (mean ± SEM; n = 3, one-way ANOVA, P< 0.0001). (G) Immunoblot of cells expressing wild-type or Rab-binding-deficient LRRK2 treated with LLOME or nigericin (1mM or 20uM respectively for 2 h) and probed for pRab10 (T73). (H) Quantification of pRab10 (T73) in wild-type and Rab-binding-deficient LRRK2 cells (mean ± SEM; n = 3, one-way ANOVA, P= 0.0008).

Having defined a Rab-dependent mechanism that links lysosomal swelling to LRRK2 activation, we next asked whether this pathway operates uniquely in response to osmotic imbalance or represents a broader principle that extends to other lysosomal perturbations, including those that trigger CASM and GABARAP lipidation. We therefore compared the ClC-7–dependent swelling pathway with the CASM–GABARAP pathway, which functions in response to lysosome damage, deacidification, or STING activation (Bentley-DeSousa, Clegg, et al., 2025). Stimuli that engage CASM, including LLOME and nigericin, continued to activate LRRK2 in *Clcn7*-KO cells (Figs. 2G–H, confirming that distinct upstream mechanisms can independently communicate to LRRK2. However, PIKfyve-induced activation was unaffected by perturbations that disrupt CASM-dependent LRRK2 activation, including ATG16L1 knockout, expression of the CASM inhibitor SopF, or GABARAP knockout (Fig. 5A–F). Conversely, the Rab-binding–deficient LRRK2 mutant failed to respond to either PIKfyve inhibition or CASM-inducing stimuli (LLOME, nigericin, and STING activation;Figs. 5G–H, S4A-B). Together, these results demonstrate that while swelling- and CASM-mediated perturbations activate LRRK2 via distinct upstream mechanisms, both converge on Rab-dependent recruitment and activation of the kinase. Lysosome swelling causes robust Rab accumulation that bypasses the need for GABARAP, whereas under conditions of lysosome deacidification that lead to CASM activation, GABARAP scaffolding becomes essential for full LRRK2 activation.

## Discussion

Our results define how LRRK2 senses and integrates distinct perturbations of lysosome physiology through coordinated interactions with Rab GTPases and GABARAP. By combining biochemical, genetic, and biophysical approaches, we identified osmotic swelling as a trigger that activates LRRK2 through a Rab-dependent mechanism distinct from, but mechanistically convergent with, the CASM–GABARAP pathway engaged by lysosome damage and deacidification (Bentley-DeSousa, Clegg, et al., 2025; Bentley-DeSousa, Roczniak-Ferguson, et al., 2025; Eguchi et al., 2024; Huang et al., 2025). These findings reveal that LRRK2 activation is not limited to a single type of lysosomal perturbation but instead arises from the integration of multiple inputs that reflect both the mechanical and chemical state of lysosomes. This integrated model provides a framework for understanding how LRRK2 is engaged downstream of lysosomal dysfunction to enable homeostatic responses while also suggesting how exaggerated or chronic activation of these same pathways may contribute to Parkinson’s disease pathogenesis through LRRK2-dependent mechanisms.

### Rab and GABARAP Interactions Allow LRRK2 to Respond to Osmotic and Membrane Stresses at Lysosomes

Our data supports a model in which LRRK2 activation arises from the integration of multiple upstream signals that report on lysosomal status. When PI(3,5)P_2_ levels fall due to PIKfyve inhibition, the resulting disinhibition of ClC-7 drives chloride influx and osmotic expansion of lysosomes. This swelling increases the membrane association of multiple Rab GTPases, including Rab8A, Rab10, Rab12, Rab29, and Rab32, creating a high-density Rab platform that recruits and activates LRRK2 independently of GABARAP. Under other stress conditions, such as deacidification arising from lysosome membrane damage or proton channel activation, CASM-mediated conjugation of GABARAP to single membranes provides an additional recruitment surface that cooperates with Rabs to engage LRRK2. Thus, Rab GTPases act as a central hub linking diverse lysosomal cues to LRRK2 activation, while GABARAP functions as a conditional cofactor that becomes particularly important when Rab enrichment is limited. This layered mechanism enables LRRK2 to interpret lysosome osmotic, membrane damage and acidification signals, integrating them into a unified response that supports downstream cellular responses. By placing LRRK2-Rab interactions and the regulation that they confer into functional contexts, our results complement previous in vitro studies of LRRK2-Rab interactions, identification of distinct Rab binding sites in LRRK2 and more general roles for specific Rabs in the regulation of LRRK2 (Alessi & Pfeffer, 2024; Dhekne et al., 2023; Kalogeropulou et al., 2020; Kuwahara et al., 2016; Liu et al., 2018; McGrath et al., 2021; Purlyte et al., 2018; Vides et al., 2022; Wang et al., 2023). In the future, it will be of interest to more narrowly define the contributions of individual Rabs to the responses of LRRK2 to distinct stimuli across cell types. However, such efforts may be complicated by the multiplicity of LRRK2-Rab interactions and potential redundancy of different Rabs. Such factors may explain observations that siRNA-mediated knockdown and over-expression experiments have previously revealed roles for specific Rabs in LRRK2 activation that were not as clearly evident when tested by complete KO of the target Rabs (Dhekne et al., 2023; Kalogeropulou et al., 2020).

### Convergent Rab and GABARAP Mechanisms Link Lysosomal Stress to LRRK2 Activation and Parkinson’s Disease Risk

Under physiological conditions, transient activation of LRRK2 at swollen or partially damaged lysosomes may facilitate localized repair, remodeling of membrane trafficking, or clearance of osmolytes, thereby restoring normal organelle function (Bonet-Ponce et al., 2020; Herbst et al., 2020). The requirement for coincident Rab and GABARAP engagement ensures that LRRK2 activation is spatially restricted to lysosomes experiencing specific combinations of mechanical and chemical stress, preventing unnecessary or diffuse kinase activity elsewhere in the endolysosomal system. The dual requirement for Rab and GABARAP interactions in the activation of LRRK2 would furthermore prevent its activation at growing autophagosomes where GABARAPs but not Rabs are abundant (Nguyen & Lazarou, 2022). However, genetic or environmental conditions that enhance LRRK2 kinase activity, increase lysosomal Rab accumulation, or chronically perturb lysosome osmotic or membrane integrity could shift this finely tuned system toward persistent activation. Candidates for engaging these pathways in the context of Parkinson’s disease pathogenesis include risk genes such as Rab32 and VPS35 that are known to activate LRRK2 and VPS13C that promotes the repair of damaged lysosomes (Gustavsson et al., 2024; Hop et al., 2024; Mir et al., 2018; Wang et al., 2025). The coupling of ClC-7 activity to LRRK2 suggests that lysosomal ionic and osmotic homeostasis are integral to kinase regulation. This connection aligns with evidence that other Parkinson’s disease–associated lysosomal transporters, including TMEM175 and ATP13A2, influence lysosomal pH and ion flux (Cang et al., 2015; Riederer et al., 2026; van Veen et al., 2020). Mutations in these genes cause lysosomal swelling and fragmentation, phenotypes that resemble those known to activate LRRK2, suggesting that LRRK2 may be engaged by lysosomal stresses across a broader context of Parkinson’s disease-relevant genetic perturbations. Meanwhile, trichloroethylene is an environmental risk factor for Parkinson’s disease that has been linked to lysosomes and LRRK2 hyperactivation via mechanisms that may converge on the pathways that we have defined here (De Miranda et al., 2021).

### LRRK2 as a coincidence detector of lysosome stress

Together, our research establishes LRRK2 as a coincidence detector that integrates mechanical and biochemical features of lysosomal stress through modular interactions with Rab GTPases and GABARAP. By linking osmotic swelling, membrane damage, and deacidification to a shared Rab-dependent activation mechanism, this work provides a unifying model for how LRRK2 monitors lysosomal integrity. This integrated signaling logic allows LRRK2 to selectively engage at lysosomes requiring repair while minimizing inappropriate activation under basal conditions or at other compartments where GABARAP and/or Rabs may reside. The recognition that biophysical and biochemical inputs converge on the same kinase opens new perspectives for understanding LRRK2’s physiological functions and offers testable hypotheses for how chronic or exaggerated activation of these stress pathways contributes to Parkinson’s disease pathogenesis. More broadly, this framework highlights lysosomal osmoregulation and membrane integrity as central control points in cellular stress signaling.

## Methods

### RAW 264.7 cell culture

RAW 264.7 cells were cultured in DMEM (11965–092; Thermo Fisher Scientific) supplemented with 10% FBS (16140–071; Thermo Fisher Scientific) and 1% penicillin/streptomycin (15140122; Thermo Fisher Scientific) at 37°C with 5% CO_2_, following the supplier’s instructions (ATCC). Cells were passaged using CellStripper (356230; Corning).

### Stable cell line generation

Stable cell lines were generated using a PiggyBac transposase system. Briefly, 2.5 × 10^5^ cells were plated per well in a 6-well dish. The following day, cells were transfected with Lipofectamine 2000 (11668019; Invitrogen) according to the manufacturer’s protocol, using a 1:2 ratio of PiggyBac transposon plasmid to gene-of-interest plasmid (total 1 μg DNA) (Pantazis et al., 2022). After 48 h, the medium was replaced, and after 72 h, puromycin (A11138–03; Gibco) was added at 3.5 μg/mL for 48–72 h. Cells were then washed and returned to fresh medium for recovery. Clonal populations were obtained by plating single cells into 96-well dishes and expanded for screening by immunoblotting. A detailed protocol is available at: dx.doi.org/10.17504/protocols.io.yxmvm9nr5l3p/v1. A custom human Halo-LRRK2 (Rab-binding-deficient) PiggyBac vector was obtained from VectorBuilder, will be made available through Addgene: pPB-EF1A-HALO-hLRRK2; Addgene_229738, pPB-EF1A-HALO-hLRRK2 (Rab binding mutant/3xRabmut K17/18A, E240R, K439E); Addgene_249537.

### Genome-edited cell lines

*Clcn7* knockout (KO) RAW 264.7 cells were generated using the Synthego CRISPR Gene Knockout V2 Mouse Kit. Briefly, 2.5 × 10^5^ cells were plated in a 6-well dish and transfected the next day with ribonucleoprotein complexes composed of recombinant Cas9 (Synthego CRISPR Gene Knockout V2) and gene-specific sgRNAs using Lipofectamine CRISPRiMAX (CMAX00003; Thermo Fisher Scientific). After 48 h, the medium was changed, and after 72 h, single cells were plated into 96-well dishes to establish clonal populations. Following clonal expansion, genomic DNA was extracted using QuickExtract, and PCR was performed using primers flanking the guide RNA cut sites. PCR products were resolved on 1% agarose gels to confirm gene disruption. A detailed protocol is available at: dx.doi.org/10.17504/protocols.io.5jyl88wj6l2w/v1.

### iPSC-Microglia Differentiation

An established iPSC line (WTC11) that was engineered for efficient doxycycline-inducible microglia differentiation was kindly provided by Li Gan (Weill-Cornell) and differentiated via their established protocol (Drager et al., 2022).

### Generation of SPIONs

Superparamagnetic iron oxide nanoparticles (SPIONs) were prepared following an established protocol (https://dx.doi.org/10.17504/protocols.io.eq2lyn69pvx9/v1). Briefly, 10mL of 1.2 M FeCl_2_ (220299; Sigma-Aldrich) and 10mL of 1.8 M FeCl_3_ (157740; Sigma-Aldrich) were combined under stirring, followed by the slow addition of 10 ml 30% NH_4_OH (320145; Sigma-Aldrich) over 5 min. The resulting particles were washed three times with 100 ml water, resuspended in 80mL 0.3 M HCl (9535; J.T. Baker), and stirred for 30 min. Dextran (4 g; D1662; Sigma-Aldrich) was then added and stirred for 30 min. The particles were dialyzed against H_2_O for ≥2 days with multiple water changes, centrifuged at 26,900 g for 30 min to remove aggregates, and stored at 4°C. A detailed protocol is available at: dx.doi.org/10.17504/protocols.io.eq2lyn69pvx9/v1.

### SPION-mediated lysosome purification

A 90% confluent 15-cm dish was split into 8, 10cm dishes. The following day, cells were incubated for 1 h with 10 ml of fresh medium containing 5% SPIONs and 5 mM HEPES (pH 7.4, 15630–080; Thermo Fisher Scientific). Dishes were washed twice with PBS and incubated in fresh medium for 2 h to allow SPION washout. All subsequent steps were performed on ice. Cells were washed, scraped in PBS, and pelleted at 300 g for 5 min at 4°C. Pellets were resuspended in 1mL ice-cold HB buffer (5 mM Tris base [AB02000–05000; American Bio], 250 mM sucrose [S0389; Sigma-Aldrich], 1 mM EGTA [E4378; Sigma-Aldrich], pH 7.4) supplemented with protease (cOmplete Mini, EDTA-free; Roche) and phosphatase inhibitors (PhosSTOP; Roche). Cells were homogenized with 50 strokes in a Dounce homogenizer (DWK Life Sciences Wheaton, 06–434) and centrifuged at 800 g for 20 min at 4°C. LS magnetic columns (130042401; Miltenyi Biotec) were equilibrated with 2.5 ml HB buffer on a QuadroMACS separator (130–091-051; Miltenyi Biotec). The supernatant was applied to the columns, and the flow-through was reapplied once. Columns were washed with 3mL HB buffer, removed from the magnet, and lysosomes were eluted in 2.5mL HB buffer into ultracentrifuge tubes. Eluates were centrifuged at 55,000 rpm for 10 min at 4°C using a Beckman-Coulter TLA-100.3 rotor. Pelleted lysosomes were resuspended in ~50 μl HB buffer for immunoblotting. A detailed protocol is available at: dx.doi.org/10.17504/protocols.io.e6nvwbk32vmk/v1.

### Induction of sucrosomes

For osmotic swelling experiments, 5 × 10^5^ cells were seeded per well in a 6-well dish. The following day, cells were washed with PBS and incubated in DMEM supplemented with 50 mM sucrose for 16 h at 37°C. Cells were imaged using an EVOS M5000 microscope and harvested for immunoblotting. A detailed protocol is available at: dx.doi.org/10.17504/protocols.io.14egnry66l5d/v1.

### Immunoblotting

For immunoblot analysis, 1 × 10^6^ cells were seeded per well in a 6-well dish. The next day, cells were washed twice with ice-cold PBS (1.1 mM KH_2_PO_4_, 155.2 mM NaCl, 3 mM Na_2_HPO_4_) and scraped into 50 μl ice-cold lysis buffer (50 mM Tris base, 150 mM NaCl, 1% Triton X-100, 1 mM EDTA) supplemented with protease (cOmplete Mini, EDTA-free; Roche) and phosphatase inhibitors (PhosSTOP; Roche). Lysates were centrifuged at 14,000 rpm for 8 min at 4°C to remove insoluble material, and protein concentrations were determined using the Coomassie Plus Protein Assay (23236; Thermo Fisher Scientific). Lysate supernatants were mixed 1:1 with Laemmli buffer (80 mM Tris-HCl, pH 6.8, 25.3% glycerol, 2.67% SDS, bromophenol blue, and 6.187% freshly added β-mercaptoethanol) and heated at 95°C for 3 min. Typically, 20 μg protein per sample was resolved on 4–15% Mini-PROTEAN TGX Stain-Free precast gels (Bio-Rad) in standard Tris-glycine-SDS buffer. For lysosome fractions, 10 μg total lysate and 1 μg purified lysosome sample were loaded. Proteins were transferred to 0.45-μm nitrocellulose membranes (1620115; Thermo Fisher Scientific) at 100V for 60 min. Membranes were visualized using Bio-Rad stain-free imaging or Ponceau S, blocked in 5% nonfat milk in TBST (10 mM Tris base, 150 mM NaCl, 0.1% Tween-20), and incubated overnight at 4°C with primary antibodies diluted in 5% BSA/TBST. After three 5-min washes in TBST, membranes were incubated for 1 h at room temperature with HRP-conjugated secondary antibodies in 5% milk/TBST, washed again, and developed using either SuperSignal West Pico PLUS or SuperSignal West Femto substrates (Thermo Fisher Scientific). Images were acquired with a Bio-Rad ChemiDoc MP imaging system. Antibodies used are listed in the Key Resources Table. A detailed protocol is available at: dx.doi.org/10.17504/protocols.io.5qpvo9bmdv4o/v1. Source data for all immunoblots will be deposited in the corresponding data repository.

### Immunofluorescence and imaging

For immunofluorescence, 2 × 10^5^ cells were plated on 12-mm glass coverslips (633029; Carolina Biological Supplies) in 6 well dishes. After treatment, cells were fixed in 4% paraformaldehyde (19202; Electron Microscopy Sciences) in sodium phosphate buffer (153.56 mM Na_2_HPO_4_, 53.63 mM NaH_2_PO_4_, pH 7.3) for 30 min at room temperature. Cells were washed three times for 5 min with PBS and permeabilized/blocked in 3% BSA and 0.1% saponin (S4521; Sigma-Aldrich) in PBS for 15 min. Primary antibodies were applied overnight at 4°C, followed by three PBS washes and incubation with fluorescent secondary antibodies for 1 h at room temperature in the dark. Cells were washed again, mounted on slides (12–550-143; Thermo Fisher Scientific) using ProLong Gold Antifade Mountant (P36935; Thermo Fisher Scientific), and stored at 4°C. Images were acquired with a Nikon Ti2-E inverted microscope equipped with a Yokogawa CSU-W1 SoRa spinning-disk super-resolution module using a 60× SR Plan Apo IR oil-immersion objective. Images were processed and adjusted in FIJI (version 2.14.0/1.54f). A detailed protocol is available at: dx.doi.org/10.17504/protocols.io.36wgqd7wkvk5/v1.

### Statistical analysis

All quantitative data were obtained from at least three independent biological replicates unless otherwise indicated. Immunoblot signals were quantified using FIJI (ImageJ, version 2.14.0/1.54f). Data are presented as mean ± SEM. Statistical analyses were carried out using GraphPad Prism (10.3.1). Comparisons between two groups were performed using two-tailed unpaired Student’s *t* tests. Multiple group comparisons were analyzed by one-way ANOVA. The number of replicates (*n*), type of statistical test, and *P* values for each experiment are reported in the corresponding figure legends.

## Supplementary Material

Supplement 1

Supplement 2

## Data Availability

All data, protocols, and key laboratory materials used or generated in this study are listed in the Key Resources Table, together with their persistent identifiers (doi: 10.5281/zenodo.17597052). No new code was generated for this study. Data processing, image analysis, and visualization were performed using FIJI/ImageJ, AlphaFold, ChimeraX, CellProfiler, and GraphPad Prism.

## Significance Statement

This study reveals how LRRK2 integrates lysosomal stress signals through coordinated interactions with Rab GTPases and GABARAP. Osmotic swelling drives strong Rab-dependent activation, whereas deacidification requires CASM-mediated GABARAP lipidation as a scaffold for LRRK2 activation at lysosomes. These results define how LRRK2 activation at lysosomes is tuned across physiological and pathogenic contexts.
