# Supplementary material for: LRRK2 integrates Rab and GABARAP interactions to sense and respond to distinct lysosomal stresses
Source: bioRxiv. 2025 Nov 19:2025.11.19.689251. Preprint. [Version 1] doi: 10.1101/2025.11.19.689251 (PMC12667755; doi:10.1101/2025.11.19.689251)
Supplement: Supplement 1 [file media-1.pdf]

Supplemental Data For:

LRRK2 integrates multiple Rab and GABARAP interactions to sense and respond to distinct lysosomal stresses

Devin Clegg[1,2,3,4,6], Amanda Bentley-DeSousa[1,2,3,4,6], Agnes Roczniak-Ferguson, and Shawn M. Ferguson[1,2,3,4,5,6*]

Departments of Cell Biology[1], Department of Neuroscience[2], Program in Cellular Neuroscience, Neurodegeneration and Repair[3], Wu Tsai Institute[4], Kavli Institute for Neuroscience[5], Yale University School of Medicine, New Haven, Connecticut 06510, USA. Aligning Science Across Parkinson's (ASAP) Collaborative Research Network, Chevy Chase, MD, 20815, USA.[6]

*Correspondence: shawn.ferguson@yale.edu

Running title: Lysosome swelling drives Rab-dependent LRRK2 activation

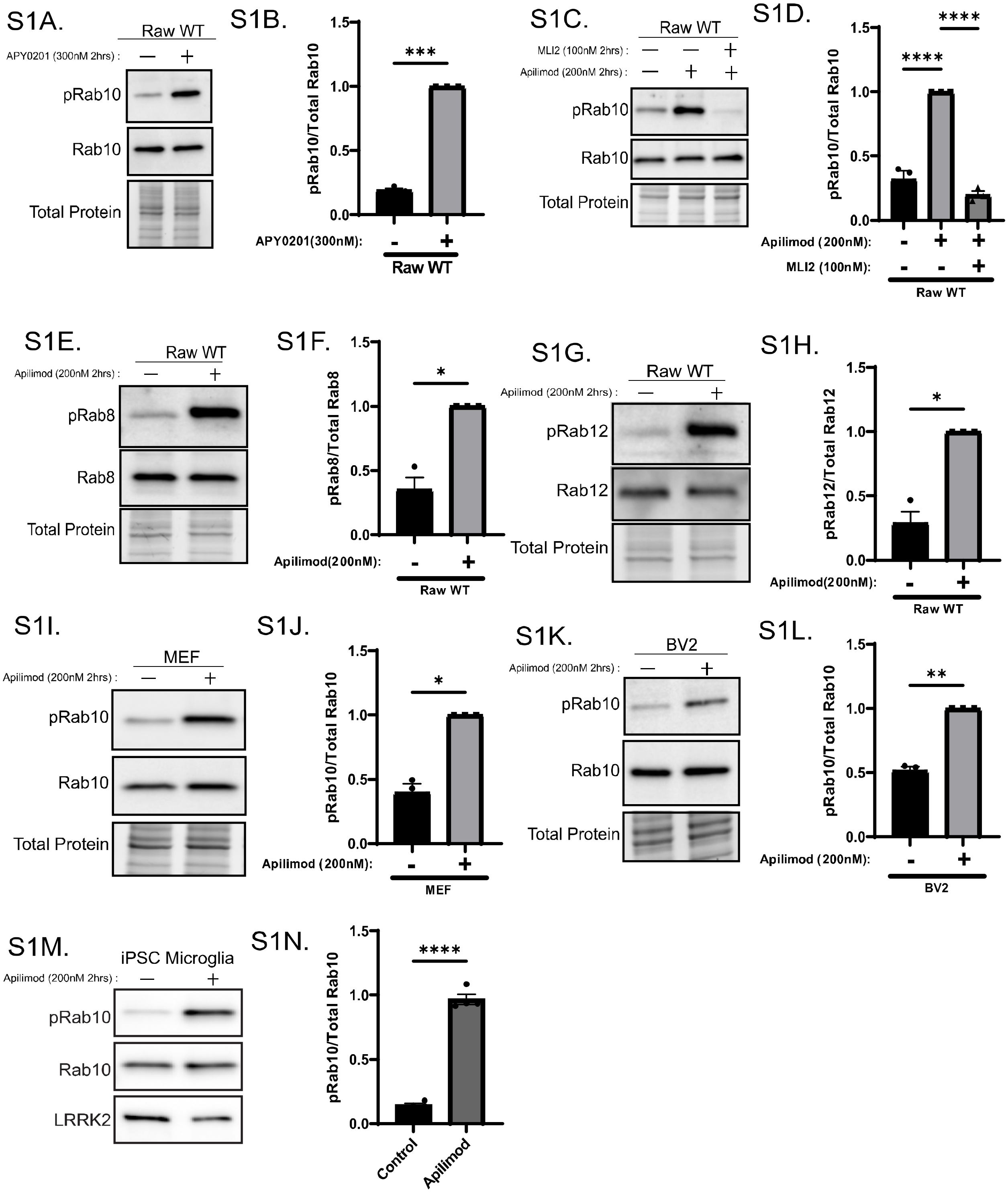

Supplemental Figure 1. PIKfyve-dependent LRRK2 activation across inhibitors and cell types. (A) Immunoblot of Raw 264.7 cells treated with APY0201 (300nM for 2 h) pRab10 (T73). (B) Quantification of pRab10 (T73) after APY0201 (mean ± SEM; n = 3, two-tailed unpaired Welch's t test, P< 0.001). (C) Immunoblot of Raw 264.7 cells

treated with apilimod (200nM, 2 h) ± MLi-2 (100nM, 2 h) and probed for pRab10 (T73). (D) Quantification of pRab10 (T73) after apilimod ± MLi-2 (mean ± SEM; n = 3, one-way ANOVA, P< 0.0001). (E–H) Immunoblots and quantifications of pRab8 (T72) and pRab12 (S106) after apilimod (mean ± SEM; n = 3, two-tailed unpaired Welch's t test, P= .021, P= .015, respectively). (I–L) Immunoblots and quantifications of MEFs and BV-2 microglia showing elevated pRab10 (T73) after apilimod (mean ± SEM; n = 3, two-tailed unpaired Welch's t test, P= .012, P= .003, respectively). (M–N) Immunoblots and quantifications of pRab10 (T73) in iPSC-derived microglia (mean ± SEM; n = 4, two-tailed unpaired Welch's t test, P< 0.0001).

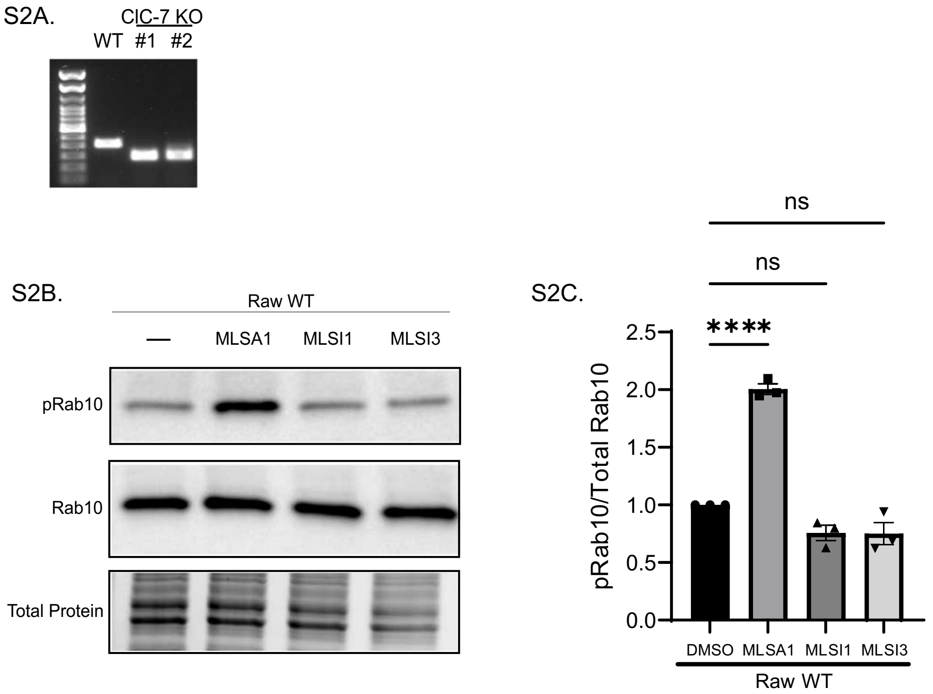

Supplemental Figure 2. *Clcn7* knockout validation and TRPML1 modulation of LRRK2 activity. (A) 1% agarose gel showing PCR analysis that confirms targeted deletions in Clcn7 KO Raw 264.7 clones. (B) Immunoblot analysis of cells treated with TRPML1 agonist ML-SA1 or inhibitors ML-SI1 and ML-SI3 (10uM, 20uM, and 25uM, respectively) and probed for pRab10 (T73). (C) Quantification of pRab10 (T73) after ML-SA1, ML-SI1, or ML-SI3 (mean ± SEM; n = 3, one-way ANOVA, $P < 0.0001$).

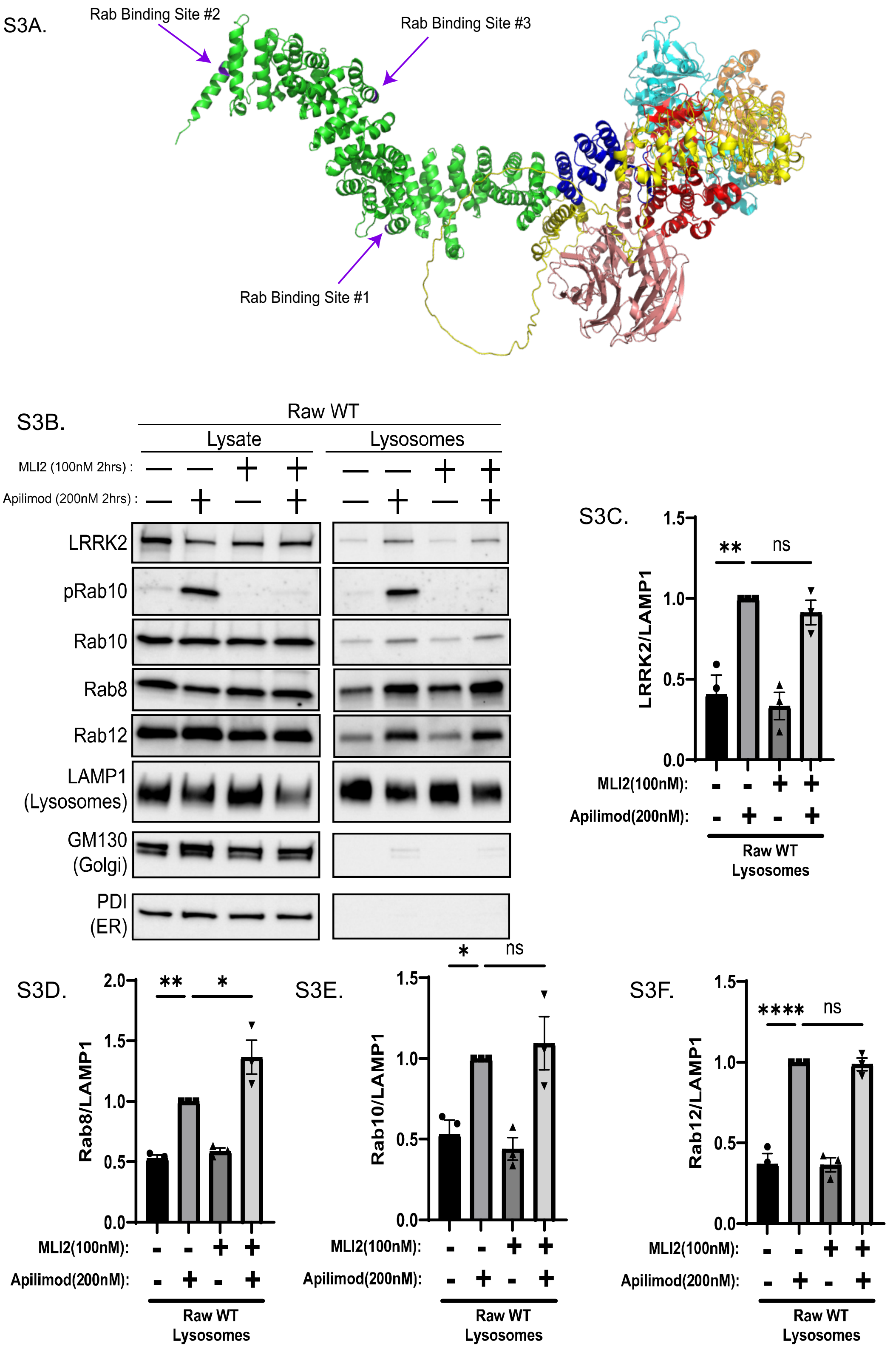

Supplemental Figure 3. LRRK2 Rab-binding mutant schematic and lack of role for LRRK2 kinase activity in LRRK2 and Rab accumulation at lysosomes following

PIKfyve inhibition. (A) AlphaFold-predicted LRRK2 structure showing three Rab-binding regions (purple arrows). (B) Immunoblot analysis of lysates and lysosomes isolated from wild-type cells treated with apilimod (200nM, 2 h) ± MLi-2 (100nM, 2 h). (C–F) Quantification of lysosomal LRRK2 and Rabs (Rab8, Rab10, Rab12) after MLi-2 ± apilimod (mean ± SEM; n = 3, two-tailed unpaired Welch's t test, $P = 0.0001$, $P = 0.0035$, $P < 0.0001$, respectively).

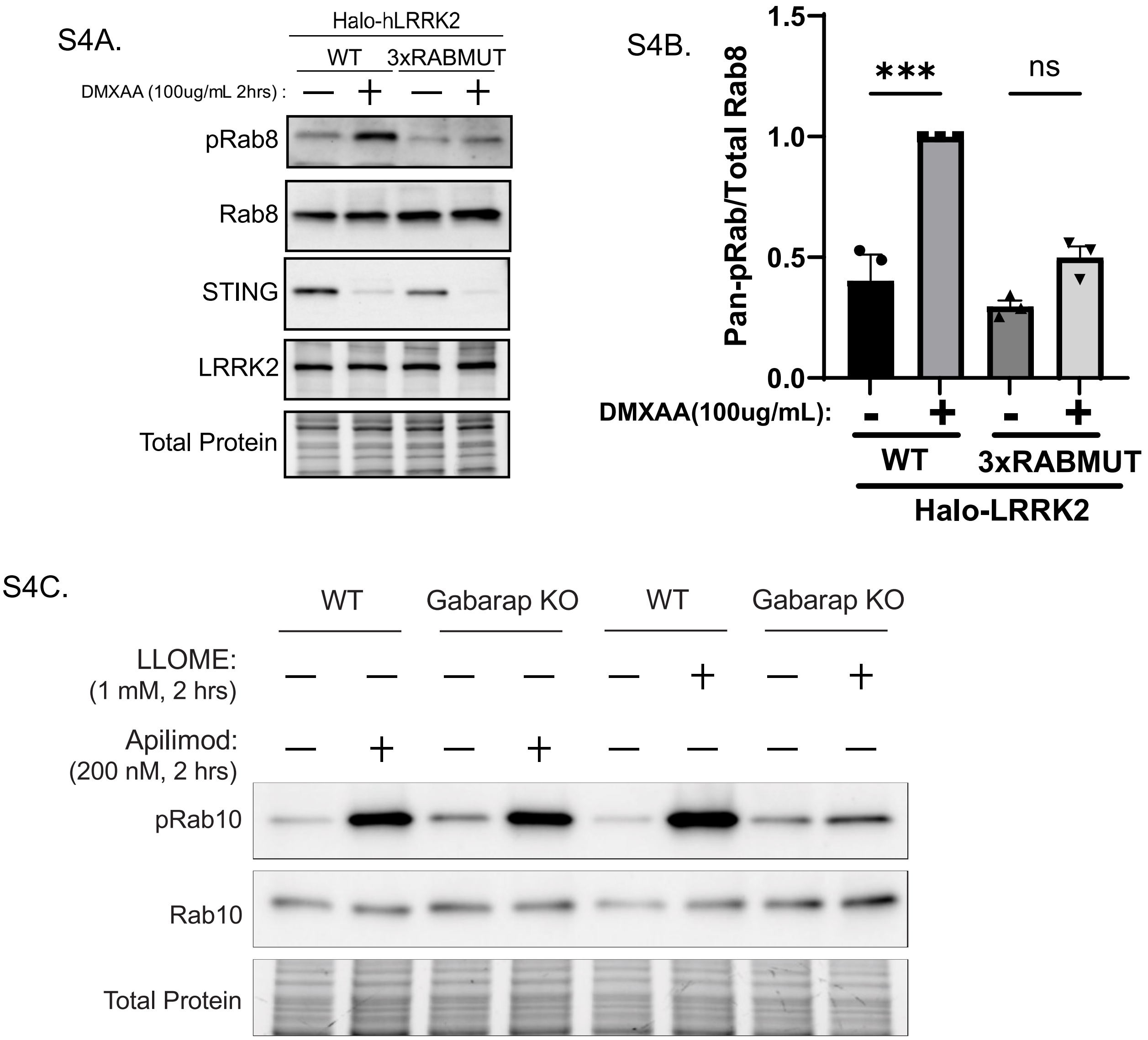

Supplemental Figure S4. Roles of Rab and GABARAP binding in LRRK2 responses to CASM-inducing stimuli. (A) Immunoblot analysis of Raw 264.7 cells stably expressing wild-type or Rab-binding-deficient LRRK2 treated with the STING agonist DMXAA (100ug/mL, 2h) and probed for pRab10 (T73). (B) Quantification of pRab10 (T73) in wild-type and Rab-binding-deficient LRRK2 cells (mean ± SEM; n = 3, two-tailed unpaired Welch's t test, P= 0.0001). (C) Immunoblot analysis of wildtype and Gabarap KO Raw 264.7 cell treated with either apilimod (200nM, 2h) or LLOME (1mM, 2h).
