# Supplementary material for: LRRK2 integrates Rab and GABARAP interactions to sense and respond to distinct lysosomal stresses
Source: bioRxiv. 2025 Nov 19:2025.11.19.689251. Preprint. [Version 1] doi: 10.1101/2025.11.19.689251 (PMC12667755; doi:10.1101/2025.11.19.689251)
Supplement: Supplement 2 [file media-2.pdf]

Key Resource Table Devin C.

RESOURCE TYPE	RESOURCE NAME	SOURCE	IDENTIFIER	NEW/REUSE	ADDITIONAL INFORMATION
**Antibody**	LRRK2	Abcam	AB_2713963	REUSE	
**Antibody**	STING	Cell Signaling Technologies	AB_2732796	REUSE	
**Antibody**	Rab10	Cell Signaling Technologies	AB_10828219	REUSE	
**Antibody**	P-Rab10 T73	Abcam	AB_2884876	REUSE	
**Antibody**	Rab12	Santa Cruz	AB_3101762	REUSE	
**Antibody**	P-Rab12 S106	Abcam	AB_2884880	REUSE	
**Antibody**	Rab8	Abcam	AB_188574	REUSE	
**Antibody**	P-Rab8	Abcam	AB_230260	REUSE	
**Antibody**	Rab29	Abcam	AB_256526	REUSE	
**Antibody**	Rab32	Santa Cruz	SC-390178	REUSE	
**Antibody**	LC3B	Thermo Fisher Scientific	AB_2234770	REUSE	
**Antibody**	GABARAP	Cell Signaling Technologies	AB_2798306	REUSE	
**Antibody**	LAMP1 (1D4B)	DSHB	AB_2134500	REUSE	
**Antibody**	PDI	Cell Signaling Technologies	AB_2298935	REUSE	
**Antibody**	GM130	BD Biosciences	AB_398141	REUSE	
**Antibody**	Rabbit IgG (HRP)	Cell Signaling Technologies	AB_2099233	REUSE	
**Antibody**	Mouse IgG (HRP)	Cell Signaling Technologies	AB_330924	REUSE	
**Antibody**	Rat IgG (HRP)	Cell Signaling Technologies	AB_10694715	REUSE	
**Antibody**	Biotin (HRP)	Cell Signaling Technologies	AB_10696897	REUSE	
**Antibody**	AlexaFluor 488 anti-rat	Invitrogen	AB_2535794	REUSE	
**Recombinant DNA**	pPB-EF1A-HALO-hLRRK2	Bentley-DeSousa et. al., JCB, 2025	Addgene_229738	REUSE	
**Recombinant DNA**	pPB-EF1A-HALO-hLRRK2 (Rab binding mutant/3xRabmut K439E, K17/18A, E240R)	This paper	Addgene_249537	NEW	
**Recombinant DNA**	pEIF1a-Piggybac transposase	Michael Ward (NINDS)(Pantazis et al., 2022)	Addgene_172116	REUSE	
**Oligonucleotide**	CLCN7 primer 1FOR	This paper	N/A	NEW	TTACCTCCTTGGGACTCCTGT
**Oligonucleotide**	CLCN7 primer 2REV	This paper	N/A	NEW	CTCTGTTCTTTACTGCCTGCC
**Experimental model: Cell line**	RAW 264.7	WT LRRK2 parental	ATCC SC-6003	REUSE	
**Experimental model: Cell line**	RAW 264.7	LRRK2 KO	ATCC SC-6004	REUSE	
**Experimental model: Cell line**	RAW 264.7	mCherry-SopF	CVCL_D7F2	REUSE	
**Experimental model: Cell line**	RAW 264.7	GABARAP KO	CVCL_D7F0	REUSE	
**Experimental model: Cell line**	RAW 264.7	Atg16L1 KO	CVCL_D7EZ	REUSE	
**Experimental model: Cell line**	RAW 264.7	LRRK2 KO + HALO-human LRRK2	Bentley-DeSousa et. al., JCB, 2025	REUSE	
**Experimental model: Cell line**	RAW 264.7	LRRK2 KO + HALO-human LRRK2 Rab binding mutant (3xRamut)	This paper	New	
**Experimental model: Cell line**	RAW 264.7	ClC-7 KO	This paper	New	
**Protocol**	Stable Cell Line Generation – RAW 264.7	protocols.io	dx.doi.org/10.17504/protocols.io.yxmvm9nr5l3p/v1	REUSE	
**Protocol**	Genome Edited (KO) Cell Line Generation –	protocols.io	dx.doi.org/10.17504/protocols.io.dm6gp9bp5vzp/v1	REUSE	
**Protocol**	Synthesis of colloidal dextran-conjugated superparamagnetic iron nanoparticles (SPIONs)	protocols.io	dx.doi.org/10.17504/protocols.io.eq2lyn69pvx9/v1	REUSE	
**Protocol**	SPION-mediated Lysosome Purification – RAW264.7	protocols.io	dx.doi.org/10.17504/protocols.io.e6nvwbk32vmk/v1	REUSE	
**Protocol**	Protein Lysate and Immunoblotting	protocols.io	dx.doi.org/10.17504/protocols.io.5qpvo9bmdv4o/v1	REUSE	
**Protocol**	Immunofluorescence - RAW 264.7	protocols.io	dx.doi.org/10.17504/protocols.io.36wgqd7wkvk5/v1.	REUSE	
**Protocol**	Genome Edited (KO) Cell Line Generation – PCR Validation	protocols.io	dx.doi.org/10.17504/protocols.io.5jyl88wj6l2w/v1	New	
**Protocol**	Sucrosome Induction in Raw 264.7	protocols.io	dx.doi.org/10.17504/protocols.io.14egnry66l5d/v1	New	
**Dataset**	Western blot source data	Zenodo	https://doi.org/10.5281/zenodo.14291079	REUSE	
**Dataset**	Immunofluorescence source data	Zenodo	https://doi.org/10.5281/zenodo.14270007	REUSE	
**Dataset**	Alphafold structural predictions	Zenodo	https://doi.org/10.5281/zenodo.14271201	REUSE	
**Dataset**	Western blot quantifications	Zenodo	https://doi.org/10.5281/zenodo.14267280	REUSE	
**Chemical, peptide, or recombinant protein**	DMEM	Thermo Fisher Scientific	11965-092	REUSE	
**Chemical, peptide, or recombinant protein**	FBS	Thermo Fisher Scientific	16140-071	REUSE	
**Chemical, peptide, or recombinant protein**	PBS	Thermo Fisher Scientific	10010023	REUSE	
**Chemical, peptide, or recombinant protein**	Cell Stripper	Corning	25056CI	REUSE	
**Chemical, peptide, or recombinant protein**	Penicillin/Streptomicin (10,000 U/mL)	Thermo Fisher Scientific	15140122	REUSE	
**Chemical, peptide, or recombinant protein**	Puromycin	Thermo Fisher Scientific	A11138-03	REUSE	
**Chemical, peptide, or recombinant protein**	Opti-Mem	Thermo Fisher Scientific	31985062	REUSE	
**Chemical, peptide, or recombinant protein**	Lipofectamine 2000	Invitrogen	11668019	REUSE	
**Chemical, peptide, or recombinant protein**	Lipofectamine RNAiMAX	Invitrogen	2448190	REUSE	
**Chemical, peptide, or recombinant protein**	Lipofectamine CRISPRMAX	Invitrogen	CMAX00003	REUSE	
**Chemical, peptide, or recombinant protein**	DMXAA	Cayman Chemicals	14617	REUSE	
**Chemical, peptide, or recombinant protein**	MLi-2	Abcam	Ab254528	REUSE	
**Chemical, peptide, or recombinant protein**	Nigericin	Cayman Chemicals	11437	REUSE	
**Chemical, peptide, or recombinant protein**	ML-SA1	Cayman Chemicals	29958	REUSE	
**Chemical, peptide, or recombinant protein**	ML-SI1	GLPBIO	GC19764	REUSE	
**Chemical, peptide, or recombinant protein**	ML-SI3	GLPBIO	GC67784	REUSE	
**Chemical, peptide, or recombinant protein**	Saliphenylhalamide	Omm Scientific	N/A	REUSE	
**Chemical, peptide, or recombinant protein**	Bafilomycin A1	Sigma-Aldrich	SML1661	REUSE	
**Chemical, peptide, or recombinant protein**	Apilimod	Tocris	7283	REUSE	
**Chemical, peptide, or recombinant protein**	Vacuolin-1	Sigma-Aldrich	673000	REUSE	
**Chemical, peptide, or recombinant protein**	LLOME	Cayman Chemicals	16008	REUSE	
**Chemical, peptide, or recombinant protein**	Phloretin	Sigma-Aldrich	P7912-25MG	REUSE	
**Chemical, peptide, or recombinant protein**	Potassium Phosphate Monobasic	J.T. Baker	Jan-46	REUSE	
**Chemical, peptide, or recombinant protein**	Sodium Phosphate Dibasic	J.T. Baker	28-May	REUSE	
**Chemical, peptide, or recombinant protein**	Glycine	American Bio	AB00730-05000	REUSE	
**Chemical, peptide, or recombinant protein**	Tris	American Bio	AB02000-05000	REUSE	
**Chemical, peptide, or recombinant protein**	NaCl	Sigma-Aldrich	24-May	REUSE	
**Chemical, peptide, or recombinant protein**	Hydrochloric Acid	J.T. Baker	9535	REUSE	
**Chemical, peptide, or recombinant protein**	SDS	American Bio	AB01920-00500	REUSE	
**Chemical, peptide, or recombinant protein**	EDTA	Sigma-Aldrich	3690	REUSE	
**Chemical, peptide, or recombinant protein**	Triton X-100	Sigma-Aldrich	X100	REUSE	
**Chemical, peptide, or recombinant protein**	Tween-20	Sigma-Aldrich	P7949	REUSE	
**Chemical, peptide, or recombinant protein**	Glycerol	American Bio	AB00751	REUSE	
**Chemical, peptide, or recombinant protein**	Bromphenol Blue	Sigma-Aldrich	B5525	REUSE	
**Chemical, peptide, or recombinant protein**	B-mercaptoethanol	Sigma-Aldrich	M3148	REUSE	
**Chemical, peptide, or recombinant protein**	Sucrose	Sigma-Aldrich	S0389	REUSE	
**Chemical, peptide, or recombinant protein**	EGTA	Sigma-Aldrich	E4378	REUSE	
**Chemical, peptide, or recombinant protein**	HEPES (pH 7.4)	Thermo Fisher Scientific	15630-080	REUSE	
**Chemical, peptide, or recombinant protein**	DMSO	Sigma-Aldrich	D2650	REUSE	
**Chemical, peptide, or recombinant protein**	COmplete mini EDTA Free	Roche	11836170001	REUSE	
**Chemical, peptide, or recombinant protein**	PhosSTOP	Roche	4906837001	REUSE	
**Chemical, peptide, or recombinant protein**	Coomassie Plus Protein Assay Reagent	Thermo Fisher Scientific	23236	REUSE	
**Chemical, peptide, or recombinant protein**	PAGEruler Plus Prestained Protein Ladder	Thermo Fisher Scientific	26620	REUSE	
**Chemical, peptide, or recombinant protein**	Biotin Protein Ladder	Cell Signaling	7727L	REUSE	
**Chemical, peptide, or recombinant protein**	4-15% MiniPROTEAN 10-well	Biorad	4568084g	REUSE	
**Chemical, peptide, or recombinant protein**	4-15% MiniPROTEAN 12-well	Biorad	4568085	REUSE	
**Chemical, peptide, or recombinant protein**	4-15% MiniPROTEAN 15-well	Biorad	4568086	REUSE	
**Chemical, peptide, or recombinant protein**	BSA	Sigma-Aldrich	A9647	REUSE	
**Chemical, peptide, or recombinant protein**	Non-Fat Dry Milk Omniblock	American Bio	AB10109-01000	REUSE	
**Chemical, peptide, or recombinant protein**	0.45 um Nitrocellulose Membrane	Thermo Fisher Scientific	1620115	REUSE	

**Chemical, peptide, or recombinant protein**	Whatman Filter Paper	VWR	28298-020	REUSE	
**Chemical, peptide, or recombinant protein**	SuperSignal West Pico PLUS Chemiluminescence Substrate	Thermo Fisher Scientific	34580	REUSE	
**Chemical, peptide, or recombinant protein**	SuperSignal West Femto Maximum Sensitivity Substrate	Thermo Fisher Scientific	34095	REUSE	
**Chemical, peptide, or recombinant protein**	Methanol	Sigma-Aldrich	179337-4L-PB	REUSE	
**Chemical, peptide, or recombinant protein**	Ethanol	Decon Laboratories	2716	REUSE	
**Chemical, peptide, or recombinant protein**	Ampicillin	Sigma-Aldrich	A0166	REUSE	
**Chemical, peptide, or recombinant protein**	Tryptone	RPI	T600-60	REUSE	
**Chemical, peptide, or recombinant protein**	LB + Ampicillin (100 µg/mL)	Recombinant Technologies	760100	REUSE	
**Chemical, peptide, or recombinant protein**	Iron (II) Chloride	Sigma-Aldrich	220299	REUSE	
**Chemical, peptide, or recombinant protein**	Iron (III) Chloride	Sigma-Aldrich	157740	REUSE	
**Chemical, peptide, or recombinant protein**	Ammonium hydroxide (30%)	Sigma-Aldrich	320145	REUSE	
**Chemical, peptide, or recombinant protein**	Dextran	Sigma-Aldrich	D1662	REUSE	
**Chemical, peptide, or recombinant protein**	Snakeskin dialysis tubing (10,000 Mol Wt)	Thermo Fisher Scientific	68100, 10,000	REUSE	
**Chemical, peptide, or recombinant protein**	LS Columns	Miltenyi Biotec	130-042-401	REUSE	
**Chemical, peptide, or recombinant protein**	QuadroMACS Separator	Miltenyi Biotec	130-091-051	REUSE	
**Chemical, peptide, or recombinant protein**	Pierce Anti-HA Magnetics Beads	Thermo Fisher Scientific	88837	REUSE	
**Chemical, peptide, or recombinant protein**	Saponin Quilajja sp.	Sigma-Aldrich	S4521	REUSE	
**Chemical, peptide, or recombinant protein**	Paraformaldehyde	Electron Microscopy Sciences	19202	REUSE	
**Chemical, peptide, or recombinant protein**	Sodium dihydrogen phosphate monohydrate	J.T. Baker	3818	REUSE	
**Chemical, peptide, or recombinant protein**	Sodium phosphate, dibasic, anhydrous	J.T. Baker	3828	REUSE	
**Chemical, peptide, or recombinant protein**	ProLong™ Gold Antifade Mountant with DNA Stain DAPI	Thermo Fisher Scientific	P36935	REUSE	
**Chemical, peptide, or recombinant protein**	Fisherbrand™ Superfrost™ Disposable Microscope Slides	Thermo Fisher Scientific	12-550-143	REUSE	
**Chemical, peptide, or recombinant protein**	Microscope Cover Slips (12 mm)	Carolina Biological Supply	633029	REUSE	
**Chemical, peptide, or recombinant protein**	Q5 High-Fidelity 2X Master Mix	NEB	M0492S	REUSE	
**Chemical, peptide, or recombinant protein**	HIFI DNA Assembly Master Mix	NEB	E2621L	REUSE	
**Chemical, peptide, or recombinant protein**	GoTaq® Green Master Mix	Promega	M7122	REUSE	
**Chemical, peptide, or recombinant protein**	One-Shot STABL3	Invitrogen	C7373-03	REUSE	
**Chemical, peptide, or recombinant protein**	Quick Extract	Biosearch Technologies	QE09050	REUSE	
**Software/Code**	Prism 10	Graphpad	SCR_002798	REUSE	
**Software/Code**	2.14.0/1.54f	FIJI	SCR_002285	REUSE	
**Software/Code**	2.5.0a0	Pymol	SCR_000305	REUSE	
**Software/Code**	AlphaFold Server	Google Deepmind	SCR_025885	REUSE	
